# Structure and Properties of Reactively Extruded Opaque Post-Consumer Recycled PET

**DOI:** 10.3390/polym13203531

**Published:** 2021-10-14

**Authors:** María Virginia Candal, Maryam Safari, Mercedes Fernández, Itziar Otaegi, Agurtzane Múgica, Manuela Zubitur, Gonzalo Gerrica-echevarria, Víctor Sebastián, Silvia Irusta, David Loaeza, Maria Lluisa Maspoch, Orlando O. Santana, Alejandro J. Müller

**Affiliations:** 1 POLYMAT and Department of Polymers and Advanced Materials: Physics, Chemistry and Technology, Faculty of Chemistry, University of the Basque Country UPV/EHU, Paseo Manuel de Lardizabal 3, 20018 Donostia-San Sebastián, Spain; mariavirginia.candalp@ehu.eus (M.V.C.); maryam.safari@polymat.eu (M.S.); mercedes.fernandez@ehu.eus (M.F.); itziar.otaegi@ehu.eus (I.O.); agurtzane.mugica@ehu.eus (A.M.); gonzalo.gerrika@ehu.eus (G.G.-e.); 2 Chemical and Environmental Engineering Department, Polytechnic School, University of the Basque Country UPV/EHU, 20018 Donostia-San Sebastián, Spain; manuela.zubitur@ehu.eus; 3 Department of Chemical and Environmental Engineering & Instituto de Nanociencia y Materiales de Aragón INMA, University of Zaragoza, Pedro Cerbuna 12, 50009 Zaragoza, Spain; victorse@unizar.es (V.S.); sirusta@unizar.es (S.I.); 4 Networking Research Center CIBER-BBN, 28029 Madrid, Spain; 5 Centre Català del Plàstic—Universitat Politècnica de Catalunya Barcelona Tech (EEBE-UPC)-ePLASCOM Research Group, Av. d’Eduard Maristany, 16, 08019 Barcelona, Spain; alfonso.david.loaeza@upc.edu (D.L.); maria.lluisa.maspoch@upc.edu (M.L.M.); orlando.santana@upc.edu (O.O.S.); 6 IKERBASQUE, Basque Foundation for Science, Plaza Euskadi 5, 48009 Bilbao, Spain

**Keywords:** recycled opaque PET, reactive extrusion, chain extension, long-chain branching

## Abstract

The recyclability of opaque PET, which contains TiO_2_ nanoparticles, has not been as well-studied as that of transparent PET. The objective of this work is to recycle post-consumer opaque PET through reactive extrusion with Joncryl. The effect of the reactive extrusion process on the molecular structure and on the thermal/mechanical/rheological properties of recycling post-consumer opaque PET (r-PET) has been analyzed. A 1% *w*/*w* Joncryl addition caused a moderate increase in the molecular weight. A moderate increase in chain length could not explain a decrease in the overall crystallization rate. This result is probably due to the presence of branches interrupting the crystallizable sequences in reactive extruded r-PET (REX-r-PET). A rheological investigation performed by SAOS/LAOS/elongational studies detected important structural modifications in REX-r-PET with respect to linear r-PET or a reference virgin PET. REX-r-PET is characterized by a slow relaxation process with enlarged elastic behaviors that are characteristic of a long-chain branched material. The mechanical properties of REX-r-PET increased because of the addition of the chain extender without a significant loss of elongation at the break. The reactive extrusion process is a suitable way to recycle opaque PET into a material with enhanced rheological properties (thanks to the production of a chain extension and long-chain branches) with mechanical properties that are comparable to those of a typical virgin PET sample.

## 1. Introduction

Plastics are a remarkable family of materials that improve the quality of life for people worldwide, because they make life easier, more comfortable, and safer. However, waste disposal is one of the major problems faced by the environment in the plastics industry. In addition, people have become used to single-use or disposable plastic. In 2018, 29.1 million tons of plastic post-consumer waste were collected in Europe to be treated. From 2006 to 2018, the volume of plastic product waste collected for recycling increased by 32.5%, the energy recovery increased by 42.6%, and landfills decreased by 24.9% [1].

Recycling plastics is one of the many initiatives launched in Europe to turn waste into resources to create a circular economy for plastics [2,3,4,5]. The majority of plastic waste is thermoplastic polymers, which can be recycled through remelting and reforming into new objects, a process which is known as mechanical recycling. The mechanical recycling of plastics is by far the most common recycling method that prevents different environmental contamination problems: the destruction of marine life and ocean biodiversity; accumulation in landfills; and harm to humans, animals, and plants, amongst others. In the last decades, increasing interest has been focused on recycling plastic wastes, especially polyethylene terephthalate (PET).

PET is one of the most widely used thermoplastic polymers, because it is a lightweight plastic with excellent mechanical, chemical, thermal, and permeability (oxygen and carbon dioxide) properties and good dimensional stability and impact resistance [6,7,8,9]. PET is mainly used for bottles (soft drinks, juices, water, carbonated soft drinks, milk, sports and energy drinks, pharmaceutical products, cooking oils, vinegar, detergents, household chemicals, dairy products, cosmetics, salad dressings, peanut butter, mouthwash, shampoo, liquid hand soap, window cleaner, even tennis balls, etc.); packaging films; and textile fibers. However, PET is not biodegradable, but it is recyclable. The PET recycling term refers to operations that aim to recover PET that can be converted into plastics items as a substituted version of virgin PET.

On the other hand, opaque (white) PET was recently introduced as UHT (Ultra-High Temperature)-treated milk packaging (bottles). Opaque PET bottles are gradually replacing coextruded and high-density polyethylene milk bottles. The use of monolayer white PET bottles is increasing, because it provides the functionality and light protection that UHT milk requires, an advantage that other packaging options do not offer. In addition, it has the potential to achieve the sustainability aspects demanded by consumers and required by regulations. Opaque PET is a PET filled with mineral nanoparticles (titanium dioxide TiO_2_ nanoparticles) that allows reducing the bottle thickness while improving the UV stability.

Today, the recyclability of opaque PET is a problem, because recycling companies are not well-prepared to separate and recycle it, unlike what happens with transparent PET [10]. Even though there are available infrastructures for collecting and sorting PET products globally, only 20−30% of transparent PET is recycled (r-PET), mostly by mechanical recycling methods. Degradation of the molecular weight during extrusion and injection molding is one of the main problems in PET recycling [11,12,13].

Chemical recycling includes various techniques that depolymerize plastic waste with some combination of solvents, heat, pressure, and catalysts into their monomers. Therefore, chemical recycling leads to consuming more solvent, time, and energy and is only economically and environmentally sound in a few cases. Mechanical recycling is an environmentally friendly and relatively straightforward process, and for this, it is a valuable technique to recycle PET. Moreover, the process conditions used are easy to control. Some authors studied several aspects of PET mechanical recycling [14]: the maximum number of extrusion cycles of PET that can reduce its mechanical properties [15], blends with virgin PET [16,17,18], the use of chain extenders that reverse some of the damage caused by polymer chain degradation [19,20,21,22], blends with other virgin polymers [23,24,25], blends with clays [26], liquid-state polycondensation (LSP), and so on. 

An exciting point is the use of chain extenders to join polymer chain segments broken during the processing and balancing of molar mass reduction due to the degradation suffered, promoting increases in the molar mass [27]. In this way, a recovery of the mechanical properties of the r-PET could be obtained. PET is chemically and thermally degraded during its processing due to its high melting temperature, sensitivity to humidity, and number of times of reprocessing. Different types of chain extenders have been used to improve the mechanical properties that PET loses during the processing. 

For example, Cavalcanti et al. [19] used triphenyl phosphite (TPP) as a chain extender of virgin PET and r-PET. They observed that virgin PET could react more easily with TPP in comparison with r-PET. Moreover, Raffa et al. [20] studied the effect of two difunctional chain extenders: namely, 1,6-diisocyanatohexane (NCO) and 1,4-butanediol diglyc-idyl ether (EPOX), in the melting properties of a r-PET. These chain extenders affected the crystallization behavior and the mechanical properties of r-PET. 

Other authors used an epoxidic multifunctional oligomer (Joncryl) recommended for processing with condensation polymers, such as PET. It is one of the most used chain extenders with the highest industrial relevance [28,29]. For example, Duarte et al. [27] explained that the molar mass is modified, depending on the additive content. They used a PET with a 1.5% concentration of Joncryl, and it preserves chain extension capabilities to sustain reprocessing without a molar mass decrease.

Recycling opaque PET (r-PET) bottles is more challenging. Few authors have investigated this problem. Tramis et al. [30] studied the increase of the mechanical properties (tensile and fatigue life) of recycled polypropylene (rPP) by the incorporation of uncompatibilized blends with r-PET. These blends could be used to substitute r-PP for similar applications. 

This work aims to recycle opaque PET, employing reactive extrusion with Joncryl (an epoxy-based chain extender).

## 2. Materials and Methods

### 2.1. Materials 

The PET obtained from opaque bottles was supplied by Suez RV Plastiques Atlantique, Bayonne, France, under the trade name of Floreal. Post-consumer recycled PET from opaque UHT milk bottles was processed to obtain two types of materials: a homogenized recycled post-consumer opaque PET (denoted r-PET) and a reactive (modified) recycled post-consumer opaque PET (denoted REX-r-PET). Prior to each processing step, r-PET materials were dried for 4h at 120 °C in a PIOVAN hopper-dryer (DSN506HE, Venice, Italy) with a dew point of −40 °C. To obtain regular geometry pellets that allow a constant feeding condition in the subsequent manufacture processes, the original heterogeneous post-consumer PET flakes were homogenized (r-PET) by a single-screw extrusion process. In this case, an extruder with L/D = 25 (IQAP-LAP E30/25, Spain) was employed with four heating zones along the profile of the screw. The temperature profile was set to 175 (hopper zone)/195/225/245 °C (die zone) and a screw rotation speed of 50 rpm. The process was performed in a N_2_-controlled atmosphere to minimize thermooxidative degradation. The r-PET filament obtained was quenched in two room temperature water baths, dried, and then cut into pellets. Then, this material was recrystallized in an oven at 120 °C for four hours to increase the crystallinity up to 20–30%. These pellets were used to prepare the REX-r-PET.

The reactive extrusion of r-PET was performed using a corotating twin-screw extruder with L/D = 36 (KNETER-25X24D, Collin GmbH, Germany). As a reactive (chain extender) reagent, a multifunctional epoxide agent (Joncryl ADR-4400® BASF, Germany) with an epoxy equivalent weight of 485 g/mol and functionality of 14 was added (1 wt%). The temperature profile of the extruder was set to 175 (hopper zone)/215/230/235/240/245/245 °C (die zone), and the screw speed was 40 rpm, leading to residence times of 4.1 min. The process was performed in vacuum to avoid further degradation. Then, the REX-r-PET product was water-cooled, dried, and pelletized; after which, the acquired material was once again recrystallized at 120 °C for 4 h.

Unprocessed PET (virgin PET) that was used for comparison purposes was supplied by Novapet (Novapet CR) with an intrinsic viscosity of 0.80 dL/g in m-cresol. For comparison purposes, it is important to note that, contrary to r-PET and REX-r-PET, virgin PET does not contain TiO_2_ particles. 

### 2.2. Intrinsic Viscosity

The intrinsic viscosity measurements were performed using an Ubbelohde Type 1B glass capillary viscometer and the ASTM D4603-03 standard method. The samples were dried 5 hours at 100 °C under vacuum, and then, they were placed in a constant temperature bath at 30 ± 0.2 °C. They were dissolved in a phenol/1,1,2,2-tetrachloroethane (60/40 (*w*/*w*)) solution at 110 °C and 50 rpm for 30 min. When the dissolution was complete, the solutions were cooled to 30 °C, filtered, and tested. According to the solvent and temperature used in this study, the Bercowitz equation can be found in the literature [31,32] in order to calculate the number-average molecular weight, *M_n_*, and the weight-average molecular weight, *M_w_*, from the solution intrinsic viscosity [*η*]:

(1) $$\left[\eta \right]=11.66*10^{-4}{\left(M_{n}\right)}^{0.648}$$

(2) $$\left[\eta \right]=7.44*10^{-4}{\left(M_{w}\right)}^{0.648}\;$$

### 2.3. Thermogravimetry Analysis (TGA)

A thermogravimetry analysis (TGA) was performed with a PerkinElmer Thermogravimetric Analyzer TGA-8000 (Waltham, MA, USA) under air atmosphere flow. The sample was heated from 40 °C up to 800 °C at a rate of 20 °C/min. All materials were dried at 100 °C for 5 h using a vacuum-oven before TGA measurement. 

### 2.4. Transmission Electron Microscopy (TEM)

The distribution of TiO_2_ nanoparticles was determined by Transmission Electron Microscopy (TEM) analysis. The samples were first cut at room temperature with a diamond knife on a Leica EMFC 6 ultramicrotome device (Leica Geosystems AG, Unterentfelden, Switzerlan). The ultra-thin sections of 90 nm thick were mounted on 200-mesh copper grids. The samples were examined using two TEM equipments: (a) TECNAI G2-20 TWIN TEM equipped with LaB6 filament operating at an accelerating voltage of 120 kV (ThermoFisher Scientific, Waltham, MA, USA) and (b) FEI Tecnai T20 thermionic LaB6 filament (FEI, Hillsboro, OR, USA) at 200 kV. To image the lamellar morphology, a RuO_4_ solution was employed for staining; then, the samples were cut and analyzed.

### 2.5. DSC Analysis

DSC measurements were performed using a PerkinElmer 8500 Pyris model (Waltham, MA, USA) calorimeter equipped with a cooling system (Intracooler 2P), under a nitrogen atmosphere flow. The DSC was calibrated with indium (*T_m, onset_* = 156.61 °C and Δ*H_m_* = 28.71 J/g). Around 5 mg of the samples were sealed in aluminum pans. The polymers were dried for 5 h under vacuum at 100 °C to remove moisture before any measurements. For nonisothermal measurements, two types of samples were studied: (1) the original pellets of virgin PET (as received), r-PET, and REX-r-PET materials (extruded only one time) were extruded a second time (at 270 °C and 80 rpm) and repelletized (results presented in Section 3.4) and (2) the samples obtained directly from the injection molded specimens (results presented in Section 3.9); see below the conditions of injection molding. For isothermal DSC measurements, only the former samples were studied.

Nonisothermal DSC measurements were performed following this sequence: (1) A first heating was performed from 25 °C up to 290 °C. (2) The previous thermal history was erased by keeping the samples at 290 °C for 3 min. (3) Cooling down the molten sample to −20 °C at a controlled rate of 20 °C/min. (4) Holding the sample at −20 °C for 1 min to equilibrate the temperature. (5) Heating up from −20 °C to 290 °C at 20 °C/min. From these measurements, all relevant transition temperatures and enthalpies were obtained. 

Isothermal measurements were performed using the procedure recommended by Lorenzo et al. [33] in extruded pellets, as mentioned before: (1) Erasing the thermal history at 265 °C (a temperature of approximately 30 °C above the peak melting temperature of the sample) for 3 min. (2) Fast cooling to the chosen crystallization temperature at 60 °C/min. (3) Holding under an isothermal state at the chosen *Tc* for a sufficient time to complete crystallization until saturation (typically, the peak time × 3). (4) Heating from *Tc* to 265 °C at a rate of 20 °C /min. The polymers were dried for 5 h under vacuum at 100 °C to remove the moisture before the isothermal experiments. The minimum isothermal crystallization temperature (*T_c_*) employed was the lowest temperature, which did not show any melting enthalpy during the immediate subsequent heating (see the details in Reference [33]).

### 2.6. Wide Angle X-ray Diffraction (WAXS)

WAXS data were collected with a Bruker D8 Advance diffractometer (Bruker, San Jose, CA, USA) operating at 30 kV and 20 mA, equipped with a Cu tube (λ = 1.5418 Å) and a Vantec-1 PSD detector. The patterns were recorded in 2θ steps of 0.033° in the 5 ≤ 2θ ≤ 38 range. For this experiment, the samples were nonisothermally crystallized from the melt (280 °C) at a rate of 20 °C/min. Then, the samples were kept at room temperature during the WAXS experiment.

### 2.7. Rheology 

The rheological properties were investigated by small-amplitude oscillatory shear (SAOS), large-amplitude oscillatory shear (LAOS), and uniaxial extensional measurements performed on a strain-controlled rotational rheometer ARES-G2 (TA Instruments, New Castle, DE, USA). The SAOS experiments were conducted at strain amplitudes in the linear viscoelastic region covering the frequency range from ω = 100 to 0.1 Hz at the temperatures T = 260, 270, and 280 °C. The LAOS experiments were performed at T = 260 °C, and the applied range of the deformation amplitude was 10–1000% at an excitation frequency of ω = 0.1 Hz. The SAOS and LAOS experiments were conducted using a parallel plate geometry with diameter d = 12 mm and gap h ≈ 1 mm. 

Extensional experiments were conducted using an EVF fixture at extensional rates between $\dot{\varepsilon }$ = 1 and 5 s^−1^ and a maximum Hencky strain of ε_H_ = 4. The rectangular sample dimensions were: thickness T = 0.6 ± 0.1 mm, width W = 10 ± 0.05 mm, and length L = 17 ± 0.5 mm. The sample was quickly loaded onto the preheated drums of the EVF fixture in the convection oven at 260 °C. 

Each experiment was performed under a nitrogen environment to prevent the oxidative degradation of samples. The disk-shaped samples for the SAOS and LAOS measurements and the rectangular-shaped films for the extensional experiments were compression-molded under vacuum at 260 °C for 5 min.

### 2.8. Injection Molding of Samples 

Injection molding was carried out using the original pellets of virgin PET, r-PET, and REX-r-PET (extruded only one time during their production) in a Battenfeld BA-230E (Wittman, Wien, Germany) reciprocating screw injection molding machine to obtain tensile (ASTM D638, type IV, thickness 2 mm) and impact (ASTM D256, thickness 3.2 mm) specimens. The screw of the plasticization unit was a standard screw with a diameter of 18 mm, L/D ratio of 17.8, and a compression ratio of 4. The melt and mold temperature, injection velocity, and cooling time were set at 270 °C, 25 °C, 10.2 cm^3^·s^−1^, and 20 s, respectively. The specimens were left to condition for 24 h in a desiccator before analysis or testing. Materials were dried in an oven for 48–72 h at 70 °C.

### 2.9. Mechanical Properties

Tensile tests were performed in a universal testing machine (Instron 5569, Norwood, MA, USA). Young modulus (*E*), tensile strength (σ_t_), and strain at break (ε_b_) were obtained from the load–displacement curves using a crosshead speed of 10 mm/min. A minimum of five tensile specimens were tested for each reported value. 

Impact tests were performed (Ceast pendulum, ASTM D-256) on the injection-molded specimens with a cross-section of 12.7 × 3.2 mm. Notches were machined in the injection-molded bars with a depth of 2.54 mm and a radius of 0.25 mm. At least eight samples were tested to determine the average impact strength. 

## 3. Results

### 3.1. Intrinsic Viscosity

In Table 1, the values of intrinsic viscosity [*η*], weight-averaged molecular weight (*M_w_*), and number averaged molecular weight (*M_n_*) are reported. Virgin PET displays an intrinsic viscosity, *η*, of 0.702 dL/g that is somewhat lower than the value reported by NOVAPET (0.80 dL/g), the virgin PET provider. The intrinsic viscosity of the homogeneized post-consumer recycled opaque PET is substantially lower than that of virgin PET, as expected. 

Hydrolytic and thermal degradation during the recycling of PET are responsible for its reduction in molecular weight. The presence of water in the PET promotes chain scission during extrusion processing [34], resulting in shorter chains with acid and hydroxyl-ester end groups. For this reason, the material was dried (see the Experimental section) at the recommended conditions to reduce the hydrolytic degradation during processing. Thermal degradation during PET recycling also results in shorter PET chains with acid and vinyl ester end groups, which contribute to the decrease of molecular weight [35]. The reduction in intrinsic viscosity of the post-consumer recycled r-PET probably arises from a combination of thermal and mechanical degradation that can occur during extrusion. Tavares et al. [36] obtained similar results for recycled PET.

However, our REX-r-PET exhibits a higher intrinsic viscosity value (0.626 dL/g) in comparison to that of the r-PET sample (0.526 dL/g). The Joncryl chain extender can react with r-PET through its epoxy groups. Both chain extension reactions and the generation of long-chain branching are possible (as reported in [20,27,36]). As a result, an increase in the molecular weight is expected after reactive extrusion of the r-PET/Joncryl blend that leads to REX-r-PET.

### 3.2. Thermal Stability

**Figure 1:**
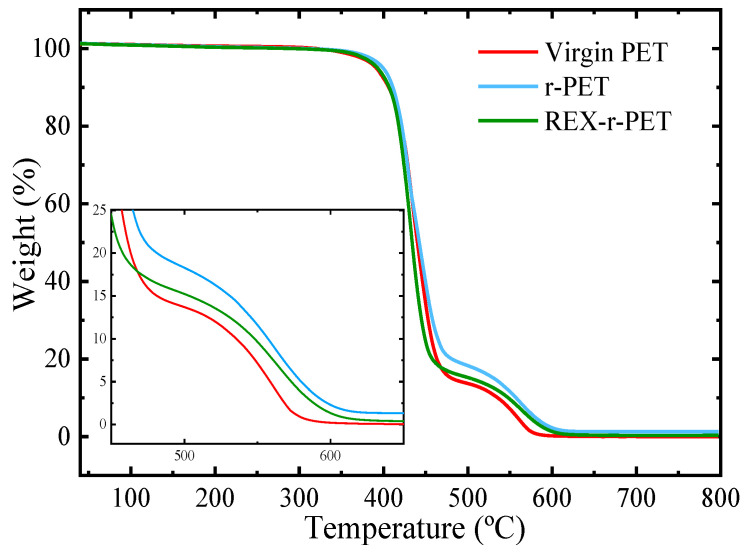
TGA traces of the indicated the PET samples recorded under air atmosphere, including an enlarged area at the 450–650 °C temperature range.

The thermal stability of virgin PET, r-PET, and REX-r-PET was studied by a thermogravimetry analysis (TGA) performed under an air atmosphere. The TGA traces are represented in Figure 1 and include two enlarged areas in order to illustrate clearly the differences between the samples in that temperature range.

The Virgin PET starts to decompose at around 326 °C (at 2% weight loss, *T_d_*_,2%_), whereas r-PET starts to decompose at 311 °C and is less stable than virgin PET (see Table 2 and Figure 1). The data in Table 2 show that the initial degradation temperatures, *T_d,_*_2%_, of r-PET were lower than that of REX-r-PET. Once again, the results are consistent with the fact that a reactive extrusion increases the molecular weight of recycled PET induced by a Joncryl addition.

All the PET samples decompose in a two-step process, a behavior that has been reported previously [37,38,39,40]. In the first weight loss step (*T_d_*_1_) with a sharp slope, at around 300–350 °C, PET chains are degraded into smaller fragments, and in the second step (*T_d_*_2_), at around 560 °C, the thermo-oxidative degradation of the small fragments occurs [41]. The remaining weight after heating to 800 °C is about 0, 2.40 ± 0.08 and 2.62 ± 0.21% for the virgin PET, r-PET, and REX-r-PET samples, respectively. This remaining weight corresponds to the percentage of TiO_2_ that is present in r-PET. Figure S1 shows that the temperatures at the maximum mass loss rate (T*_d_*_1_) for virgin PET, r-PET, and REX-r-PET are 432, 430, and 432 °C, respectively. The second step of the thermal degradation values, *T_d_*_2_, are similar for all the PET samples, at around 560 °C.

### 3.3. TEM Observations of TiO_2_ Nanoparticle Dispersion 

**Figure 2:**
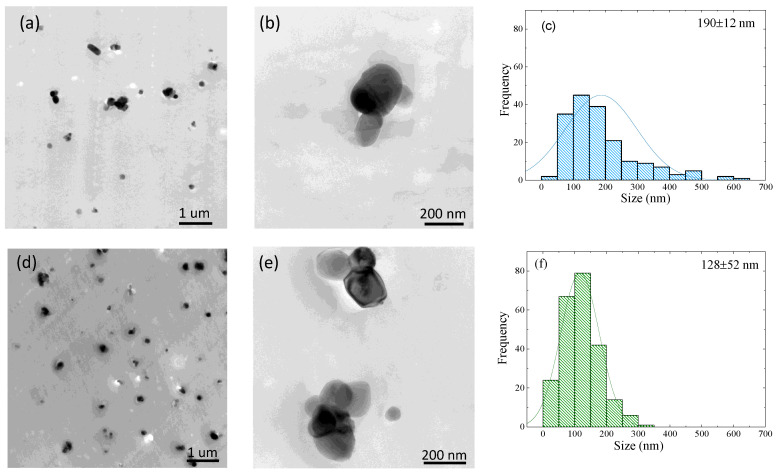
TEM images of r-PET (**a**,**b**) and REX-r-PET (**d**,**e**) samples and corresponding TiO_2_ sizes of particle distributed in the PET matrix for r-PET (**c**) and REX-r-PET (**f**). *N* > 200 particles.

The size and dispersion of TiO_2_ nanoparticles inside the recycled PET matrix were observed by TEM. Figure 2a,b and Figure 2d,e are TEM images of r-PET and REX-r-PET, respectively. For both samples, Figure 2 shows that TiO_2_ nanoparticles clusters are uniformly distributed inside the matrix. Particle aggregation into clusters can be appreciated at the higher magnification images. Figure 2c and Figure 2f shows the dispersion of TiO_2_ particles within r-PET and REX-r-PET samples. The particle size histograms (in fact, cluster sizes) in both samples were determined by ImageJ software (Version 1.48f, NIH, Bethesda, MD, USA); the mean particle size and standard deviation results are inserted in the plots. The r-PET sample shows a wider TiO_2_ cluster size distribution as compared to REX-r-PET. This is probably due to the fact that REX-r-PET is prepared by a reactive extrusion of r-PET. Therefore, it undergoes an additional extrusion step that allows breaking aggregates clusters, thereby reducing the dispersion of the cluster distribution in the matrix. However, the final average TiO_2_ cluster diameter is very similar when the errors involved in the measurements are taken into account: 190 ± 12 nm for r-PET and 128 ± 52 nm for REX-r-PET.

### 3.4. Nonisothermal Crystallization by DSC

**Figure 3:**
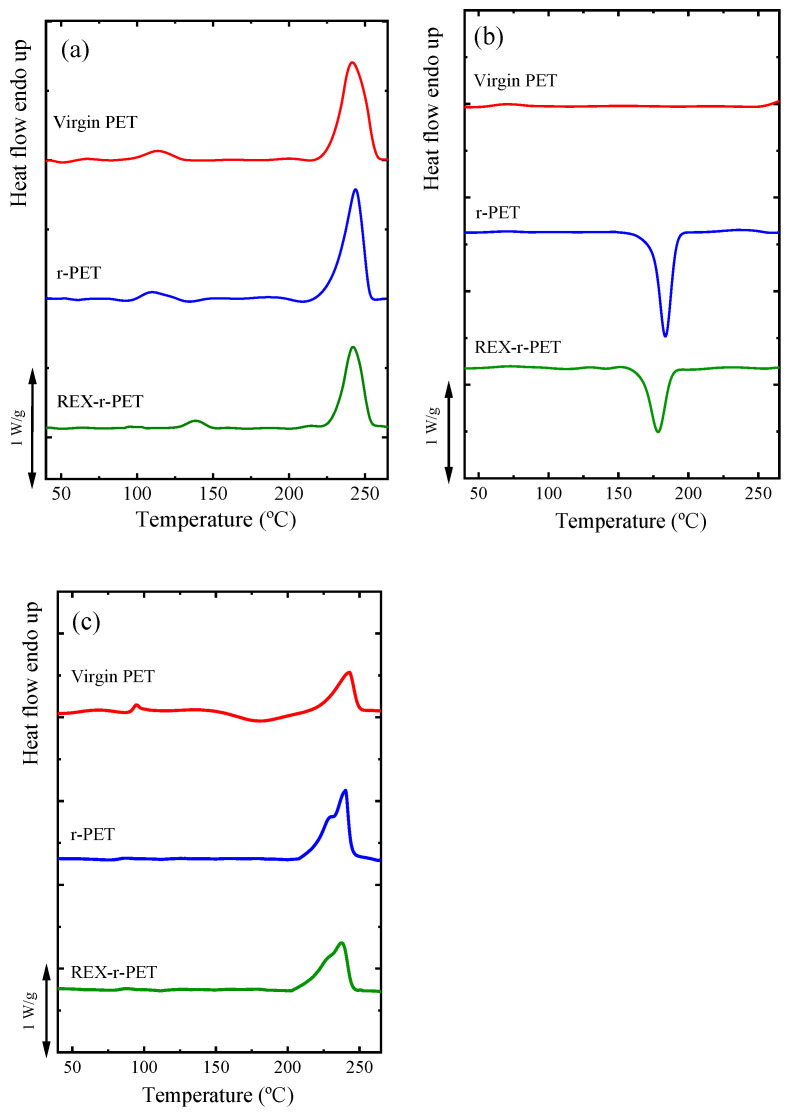
DSC scans of the PET samples: (**a**) first heating runs, (**b**) cooling runs from the melt, and (**c**) subsequent heating runs. Cooling and heating rates were 20 °C/min in all cases.

The nonisothermal crystallization and melting behavior of virgin PET and the recycled PET samples (recrystallized pellets) are presented in Figure 3a,b. The related thermal transition data, measured from the cooling and second heating scans, including the melting temperature (*T_m_*), crystallization temperature (*T_c_*), melting enthalpy (Δ*H_m_*), and crystallization enthalpy (Δ*H_c_*), are reported in Table 3.

The virgin PET is used as a reference in this work, and it does not contain TiO_2_. However, r-PET is a post-consumer material that contains PET from different sources and titanium dioxide particles. Therefore, a direct comparison is not possible in quantitative terms. 

Figure 3a shows that all samples melt at different temperatures, reflecting their thermal histories, in a range of 241–243 °C. After the thermal history is erased, the crystallization of virgin PET cannot be appreciated during cooling from the melt, as shown in Figure 3b, as expected from its well-known slow crystallization kinetics. On the other hand, both recycled PET samples are able to crystallize during cooling from the melt (Figure 3b). This could be connected to the decrease in the molecular weight and, also, to a nucleating action of titanium dioxide [28]. 

The second heating scans, in Figure 3c, show the melting endotherms for all the samples. As can be seen in Figure 3c, the r-PET and REX-r-PET samples show a slight bimodality in their melting peaks. A similar behavior was reported for PET samples containing more than 1 wt% nanoparticles [42,43]. 

The melting enthalpy values (Δ*H_m_*) of the recycled PETs in the second heating runs are higher as compared to virgin PET. The relative crystallinity *X_c_* was calculated using the following equation:

(3) $$X_{c}=\frac{\Delta H_{m}-\Delta H_{cc}}{\left(1-n\right)\Delta H_{m}^{0}}\times 100$$

where Δ*H_m_*_0_ is the melting enthalpy of 100% crystalline PET, which is reported in the literature as 140 J/g [44], Δ*H_cc_* is the cold crystallization enthalpy (detected only in virgin PET; see Figure 3c), and *n* is the quantity (%) of TiO_2_ nanoparticles [45].

### 3.5. WAXS

**Figure 4:**
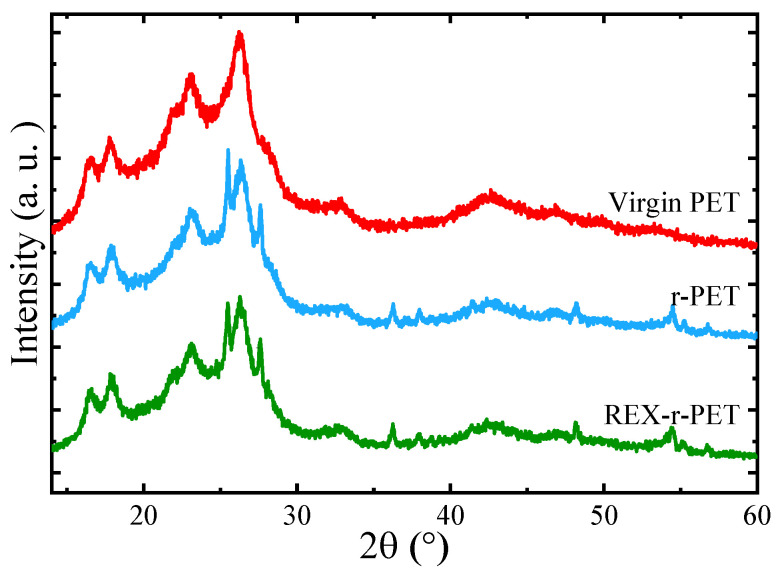
WAXS patterns of the PET samples at 25 °C after nonisothermal crystallization from the melt at a rate of 20 °C/min.

Figure 4 shows the WAXS patterns of the selected PET samples that were nonisothermally crystallized from the melt by cooling at 20 °C/min. The WAXS patterns of the three samples examined revealed a semicrystalline structure with reflections characteristic of the crystallographic planes (011), (010), (110), and (100) for scattering angles at 2θ = 16.59°, 17.81°, 23.04°, and 26.25°, respectively [46]. The unit cell of PET is triclinic with a = 4.56A, b = 5.94A, c = 10.75A, α = 98.5°, β = 118°, and γ = 112° [47]. A detailed comparison of the WAXS patterns of the virgin PET, r-PET, and REX-r-PET samples indicates that the main reflections of the above-mentioned crystallographic planes do not shift in the angular position. The crystal unit cell of PET remains identical for all the samples. 

WAXS patterns of r-PET and REX-r-PET show the presence of TiO_2_ nanoparticles within the PET matrix. The characteristic peaks of the anatase (101) and rutile (110) crystalline phases of TiO_2_ are located at 2θ = 25.47° and 27.59°, respectively. 

### 3.6. Overall Isothermal Crystallization by DSC

**Figure 5:**
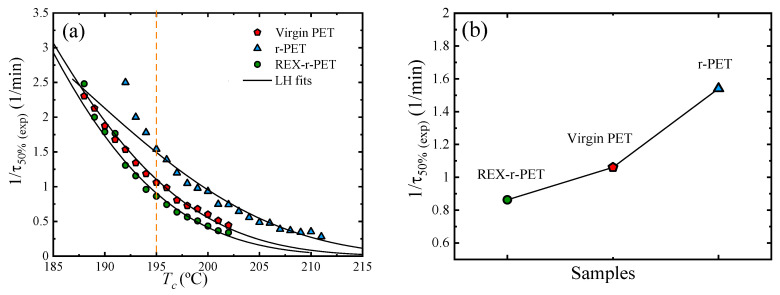
(**a**) Overall crystallization rate (*1/t*_50%_) as a function of the crystallization temperature *T_c_.* The solid lines represent fits to the Lauritzen and Hoffman theory. (**b**) Crystallization temperature values for the samples at a constant crystallization temperature of 195 °C.

Isothermal crystallization experiments performed by DSC are used to determine the overall crystallization kinetics (that comprises nucleation and growth). The inverse of the crystallization half-time (1/*τ*_50%_), which represents the overall crystallization rate as a function of *T_c_*, is shown for all the samples in Figure 5a. The overall crystallization rate decreases with the crystallization temperature (*T_c_*), indicating that, in this *T_c_* range, the overall crystallization rate is dominated by nucleation (both primary and secondary nucleation) [48]. 

According to Figure 5a, the order of the crystallization rate at any constant temperature is: r-PET > virgin PET > REX-r-PET, as illustrated in Figure 5b for a constant crystallization temperature of 195 °C. It is interesting to note that the results cannot be explained in terms of a simple difference in the molecular weights between the samples. It would be expected that, as the molecular weight decreases, the overall crystallization kinetics would increase in this molecular weight range. Comparing virgin PET with the homogenized post-consumer PET sample (r-PET), the expected behaviour is observed, as the recycled material has a lower molecular weight, which enhances its overall crystallization rate.

If the REX-r-PET sample would be constituted just by linear chains, then its overall crystallization rate should have been between virgin PET and r-PET, according to their molecular weight differences (Table 1). However, as it will be shown by the rheological measurements, the reactive extrusion of PET/Joncryl leads not only to chain extension but, also, to the production of long-chain branching. Branching interrupts the linear crystallizable sequences of the PET chains, acting as defects (which are normally forced out of the crystals and into the amorphous regions), thereby reducing the crystallization rate, as shown in Figure 5a. In this way, even though the molecular weight of the virgin PET used here is higher than that of REX-r-PET, it has a higher crystallization rate because of the differences in the chain structure between the two samples.

In the previous analysis, the presence of the TiO_2_ nanoparticles has been ignored. As they are both present in r-PET and REX-r-PET, their influence should be identical in these two materials, as their amount is approximately the same (about 2.5%; Table 2), and their small clusters are well-distributed in the PET matrix. 

The Lauritzen and Hoffman model can be applied to fit the overall crystallization data obtained by DSC experiments using the following equation [49,50]:

(4) 1τ50%=1τ0exp[UR(Tc−T0)][−KgτfT(Tm0−Tc)]

where 1/*τ*_0_ is a growth rate constant, and U* is the transport activation energy that characterizes molecular diffusion across the interfacial boundary between melt and crystals (that, in this work, is taken as a constant value of 1500 cal/mol). *T_c_* is the crystallization temperature, and *T*_0_ is a hypothetical temperature at which all chain movements freeze (*T*_0_ = *T_g_* −30 °C). *T_m_*_0_ is the equilibrium melting temperature of the polymer, and R is a gas constant. $K_{g}^{\tau }$ is a constant proportional to the energy barrier for both primary and secondary nucleation. $K_{g}^{\tau }$ is given by:

(5) $$K_{g}^{\tau }=\frac{jb_{0}\sigma \sigma_{e}T_{m}^{0}}{k\Delta H_{f}}$$

where *j* is assumed to be equal to 2 for crystallization in the so-called Regime II, a regime where both secondary nucleation at the growth front and the rate of spread along the growing crystal face are comparable. The other terms in the equation are: the width of the chain *b*_0_, the lateral surface-free energy *σ*, the fold surface-free energy *σ_e_*, the Boltzman constant *k*, and the latent equilibrium heat of fusion, $\Delta H_{f}^{\;}$ [51,52,53,54].

The solid lines in Figure 5a represent fits to the Lauritzen–Hoffman theory, according to Equation (2). All the relevant parameters obtained from the L–H equation are listed in Table 4, and they are similar to those previously reported in the literature [51,52,53,54]. The obtained $\;K_{g}^{\tau }$ values were found to be equal to 3.81 × 10^5^ for virgin PET, 3.84 × 10^5^ for r-PET, and 5.91 × 10^5^ for the REX-r-PET sample. Therefore, as $K_{g}^{\tau }$ characterizes the energy barrier for secondary nucleation, there is a significant increase of the energy barrier for nucleation (both primary and secondary nucleation) in the case of the reactively extruded sample, REX-r-PET, as expected. Therefore, the L–H theory correctly predicts that the energy barrier to crystallize the REX-r-PET sample is the highest in comparison with the other PET samples employed here. Both the fold surface-free energy and the work to fold chains follow the same trend as $K_{g}^{\tau }$, as observed in Table 4, because these parameters are directly related through Equation (5).

Values obtained by fitting the L–H theory to the experimental DSC overall crystallization data. Parameter proportional to the energy barrier for the secondary nucleation ($K_{g}^{\tau }$), fold surface energy (*σ_e_*), and work done by the chain to perform a fold (*q*). *R^2^* is the correlation coefficient for the fitting of the L–H model Equation (4).

#### Fitting the DSC Isothermal Crystallization Data to the Avrami Theory

The Avrami Equation (6) can describe the overall crystallization process in polymers [33] as:

(6) $$1-Vc\left(t-t_{0}\right)=exp\left(-k{\left(t-t_{0}\right)}^{n}\right)$$

where *V_c_* is the relative volumetric transformed fraction (as a function of time), *t* is the experimental time of crystallization, *t*_0_ is the induction or incubation time, *k* is an overall crystallization rate constant, and *n* is the Avrami index. The Origin plug-in (developed by Lorenzo et al.) was employed [33] to fit the Avrami equation to the experimental data. Figure S2 shows a representative fit of the Avrami theory for the crystallization of the r-PET sample at 200 °C (Table S1).

Figure S2a shows the Avrami plot derived from Eq. 6 that is linearized by the logarithmic scale of the axis, within a 3–20% conversion range that corresponds to the early stage of primary crystallization, before any spherulitic impingement takes place. The Avrami parameters are included in the plot for the indicated example. The normalized experimental heat flow data is well-modeled by the Avrami fit using the obtained values from Equation (6), and a comparison between experimental data and predictions from the Avrami equation is plotted in Figure S2b.

**Figure 6:**
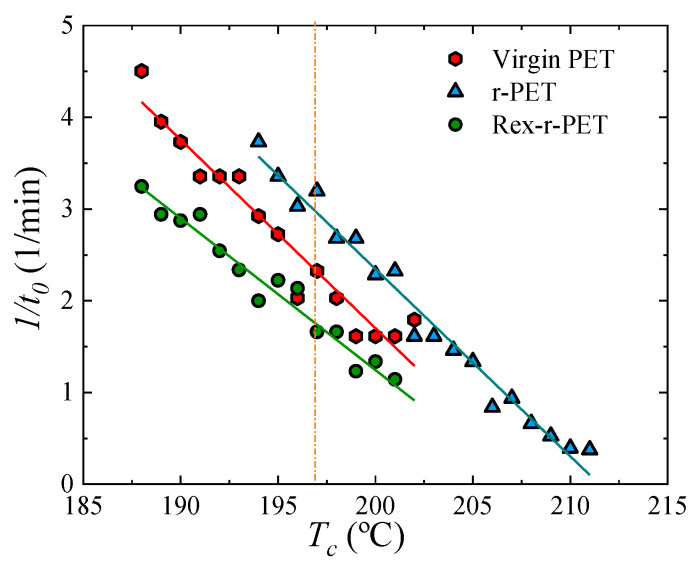
Primary nucleation rate obtained by DSC for the samples isothermally crystallized from the melt as a function of *T_c_*.

Figure 6 shows the inverse of the induction or incubation time for the primary nucleation (*1/t*_0_) from the melt state before any crystallization has started, as a function of *T_c_*. The value 1/*t*_0_ is proportional to the primary nucleation rate. As seen in Figure 6, in general, for all the samples, the nucleation rate decreases with the crystallization temperature, as in the temperature range explored, where the primary nucleation is not affected by diffusion contributions. When a constant crystallization temperature is fixed, the order of the nucleation rate is similar to that observed for the overall crystallization rate (*1/t*_50%_) presented above. This is an indication of the importance of the primary nucleation rate as a determining factor in the final overall crystallization rate, which includes both primary nucleation and growth. The REX-r-PET sample shows a lower nucleation rate value than r-PET. This is related to the differences in both the molecular weight and chain structure. 

**Figure 7:**
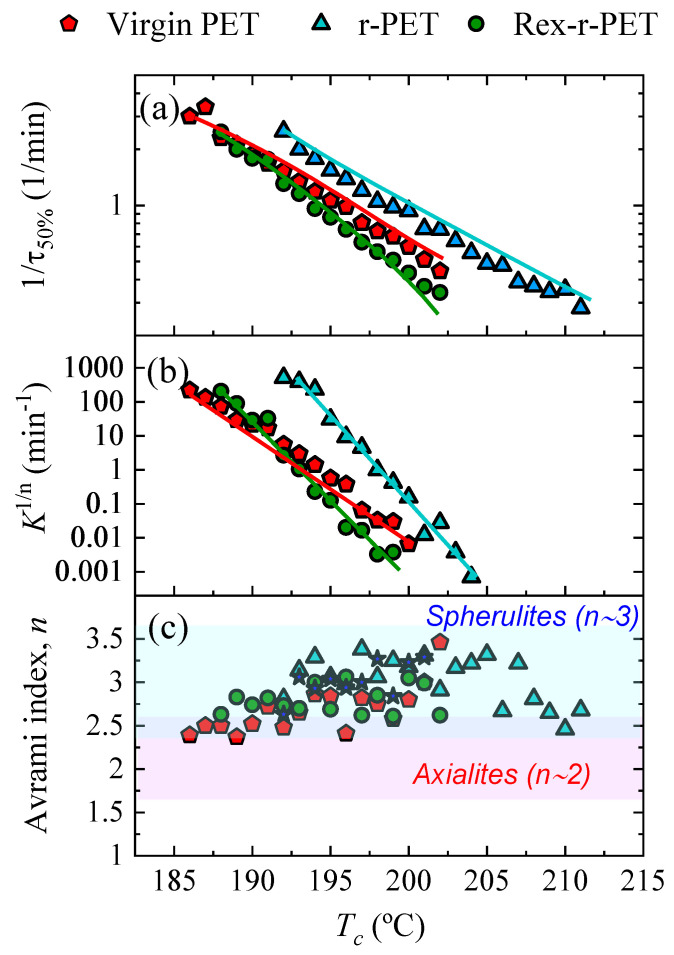
(**a**) Overall crystallization rates, indicated as the inverse of the experimentally determined half-crystallization times presented on a logarithmic scale, (**b**) normalized crystallisation constant obtained from the Avrami model presented on a logarithmic scale, and (**c**) Avrami index for the indicated PET samples as a function of the crystallization temperature, *T_c_*. Solid lines in (**a**) and (**b**) correspond to the fit of the Lauritzen–Hoffman (L–H) theory.

Figure 7 summarizes the kinetic parameters of the overall crystallization as a function of the crystallization temperature for the three PET samples examined here. In Figure 7a, the inverse of the experimental half-crystallization (*1/τ*_50%_) data is plotted versus *T*_c_. The solid lines in Figure 7a correspond to the Lauritzen–Hoffman (L–H) fitting. The *k*^1/*n*^ values were calculated from the Avrami theory parameter by elevating the k (isothermal overall crystallization rate constant) to 1/*n* (1/Avrami index), so that consistent units are obtained (min^−1)^ and their values compared. The *k*1/*n* values are plotted versus *T_c_* in Figure 7b. The solid lines in Figure 7b correspond to the fit of the Lauritzen–Hoffman (L–H) theory. The similarity between Figure 7a,b is a consequence of the Avrami equation fitting to the experimental overall crystallization rate data, which works reasonably well up to approximately 50% conversion. The fastest crystallization of the lowest molecular weight sample (r-PET) can be clearly appreciated, as already discussed above.

Figure 7c presents the values of the Avrami index *n* as a function of *T_c_*. The values fluctuate between 2.5 and 3.5, which are characteristic of instantaneously nucleated (*n* approximately equal to 3) and sporadically nucleated (*n* approximately equal to 4) spherulites, respectively.

### 3.7. Lamellar Thickness Distribution and TEM Observations

A ruthenium tetroxide (RuO_4_) solution was used to stain the samples. The RuO_4_ atoms penetrate and stain the amorphous regions of PET, while the crystalline regions remained practically unstained. Due to this, the lamellae inside the spherulites can be clearly seen in white color in contrast with the dark amorphous interlamellar regions. 

**Figure 8:**
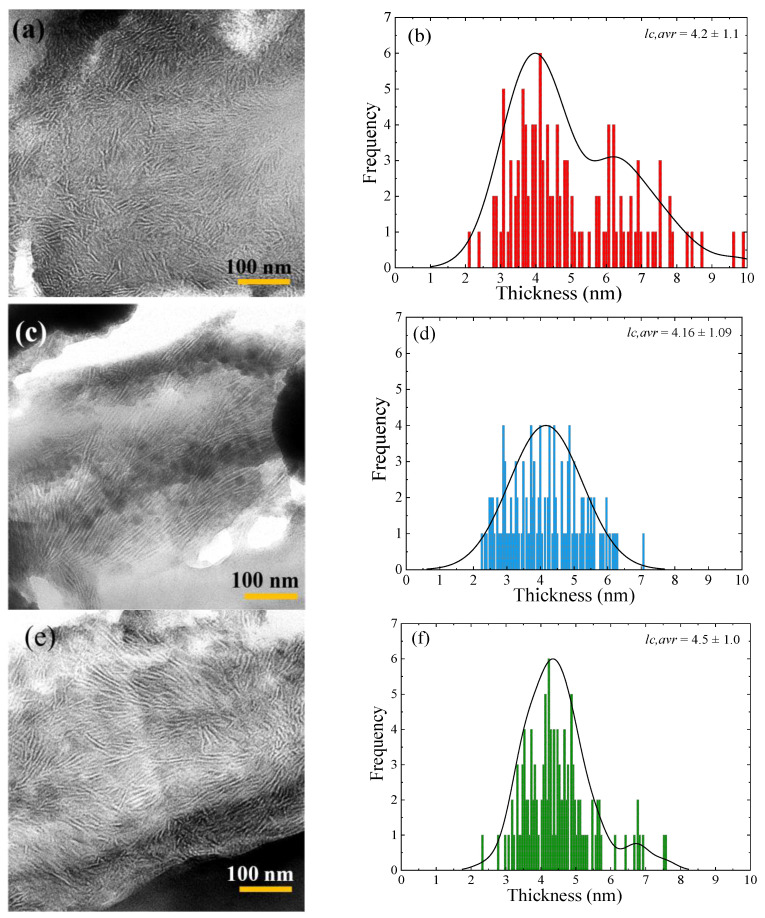
TEM micrographs of RuO_4_-stained PET samples: (**a**) virgin PET, (**c**) r-PET, and (**e**) REX-r-PET samples. Frequency distribution of lamellar thickness for (**b**) virgin PET, (**d**) r-PET, and (**f**) REX-r-PET.

Figure 8a, 8c and 8e are the corresponding TEM micrographs for the films crystallized at 200 °C for 2 h and then immediately quenched to room temperature for virgin PET, r-PET, and REX-r-PET, respectively. These TEM micrographs present well-defined stacked lamellar morphology within spherulites. 

Lamellar thickness distributions were calculated from TEM observations using Digimizer software and are presented in Figure 8b,d,f for virgin PET, r-PET, and REX-r-PET, respectively. The average lamellar thickness, *l*_c,ave_, was calculated for all the studied samples, and the values were inserted in the plots. The average lamellar thicknesses for virgin PET and REX-r-PET are bigger than that of the r-PET sample. In addition, there are two populations of lamellar thickness distribution in virgin PET and REX-r-PET, one in the range of 2–6 nm and the other one around 6–10 nm. That means the frequency distribution of the lamellar thickness has a bimodal shape in these samples. However, the r-PET sample shows only a single lamellar thickness distribution in the range of 2–6 nm.

As described before in the experimental section, the samples were isothermally crystallized at 200 °C for 2 h. Then, the samples were quenched from 200 °C to room temperature and kept at this temperature to perform the TEM observations. Probably, this bimodal distribution of lamellar thickness comes from the crystallization of the samples at 200 °C and during cooling from that temperature. First, the isothermal crystallization of the samples at 200 °C (a high crystallization temperature) produced the largest lamellae of sizes around 6-10 nm. During the quenching of the sample to room temperature, smaller-sized lamellae were formed (2–6 nm). Modified PET with the reactive recycling (REX-r-PET) method shows a similar bimodal lamellar distribution as virgin PET. However, the homogenized PET (r-PET) with a lower molecular weight shows only one population of lamellae with an average of 4.16 nm. This means that the majority of the lamellae were probably formed during cooling from 200 °C at lower temperatures. This effect can be attributed to the molecular segregation processes during crystallization that are proportional to the molecular weight. Therefore, the average lamellar size distribution curves are sensitive to PET processing (i.e., cooling conditions) and molecular weight.

### 3.8. Rheology 

#### 3.8.1. Linear Viscoelastic Data under SAOS (Small Amplitude Oscillatory Shear) 

**Figure 9:**
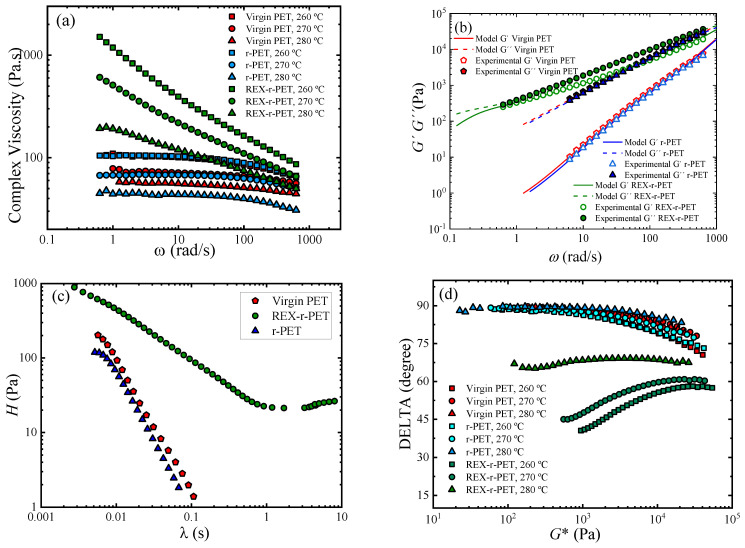
Linear viscoelastic data for virgin PET, r-PET, and REX-r-PET. (**a**) Complex viscosity at three temperatures: *T* = 260 °C, *T* = 270 °C, and *T* = 280 °C. (**b**) Moduli data obtained at *T* = 270 °C fitted to the Maxwell model. (**c**) Relaxation time spectra for virgin PET and REX-r-PET, and (**d**) van Gurp Palmen plot at temperatures *T* = 260, 270, and 280 °C for virgin PET, r-PET, and REX-r-PET.

The small amplitude oscillatory shear tests, SAOS, assume that the response of the material is in the linear viscoelastic regime, and the functions of the material, storage modulus, *G′*, and loss modulus, *G″* (as well as the derived viscoelastic parameters), determined as a function of the frequency, fully describe the material response. Since linear viscoelasticity is based on a rigorous theoretical basis [55,56,57], SAOS tests provide a very useful and convenient rheological characterization of polymers of different molecular architectures. The linear viscoelastic parameters of the three PET samples: virgin PET, r-PET, and REX-r-PET, are presented in Figure 9. 

The modified REX-r-PET sample compared to virgin PET and r-PET shows a pronounced increase in both the complex viscosity (Figure 9a) and elasticity (Figure 9b). Virgin PET and r-PET display terminal behavior and Newtonian viscosity in the studied frequency range, whereas REX-r-PET is characterized by displaying the onset of the terminal regime, which is shifted to frequencies lower than those accessible experimentally. The corresponding moduli of REX-r-PET slowly decrease with the frequency so that the quasiparallel moduli response indicates a gel-like behavior. The Newtonian plateau was not reached within the measured frequency–temperature window because of the slow relaxation, and a pronounced shear-thinning behavior was observed. 

Therefore, the viscoelasticity and chain relaxation were greatly affected by the reactive extrusion treatment. The rapid relaxation of the virgin and homogenized PET (r-PET) would be related to their linear structure that led to a Newtonian plateau and a very weak elastic response, while the slower relaxation process of REX-r-PET is probably related to the formation of larger chains (the increase of the intrinsic viscosity is reported in Section 3.1) and/or by long-chain branches (LCB) during reactive extrusion with Joncryl [58,59]. To study this behavior in more depth, the viscoelastic parameters *G′* and *G″* were evaluated in terms of a discrete relaxation spectrum modeled from the mechanical spectrum of the virgin PET and REX-r-PET samples using TA Instruments TRIOS® software by applying the following equations:

(7) $$G^{\prime }\left(\omega \right)=\int_{0}^{\infty }H\left(\lambda \right)\frac{{\left(\omega \lambda \right)}^{2}}{1+{\left(\omega \lambda \right)}^{2}}\frac{d\lambda }{\lambda }$$

(8) $$G^{\prime \prime }\left(\omega \right)=\int_{0}^{\infty }H\left(\lambda \right)\frac{\omega \lambda }{1+{\left(\omega \lambda \right)}^{2}}\frac{d\lambda }{\lambda }$$

The calculated average relaxation times, defined as  λ¯=∑iGiλi2∑iGiλi, were very different for both samples: *λ* Virgin PET = 0.015 s, *λ* r-PET = 0.010 s, and *λ*REX-r-PET = 7 s. 

Figure 9c shows the comparison of the relaxation spectrum *H*(*λ*) of each PET, where λ represents the relaxation time. The main relaxation motion of Virgin PET and r-PET was less than one second, which would be attributed to a reptation mechanism. That is, the main relaxation mechanism is not influenced by the homogenization extrusion process or the presence of TiO_2_ particles that characterize the r-PET sample. On the contrary, the REX-r-PET spectrum distinguishes the contribution of a rubbery state at times longer than one second, where strong entanglements would block the motion and enlarge the spectrum. The rubbery state, which is very similar to that recently reported by Ge et al. [60] for LCB-PET, would indicate the simultaneous relaxation processes given by short, long, and LCB (long chain-branched) chains and could be understood in terms of a branched-chain backbone. 

Branched polymers are particularly thermorheologically sensitive. For example, branched polymers exhibit higher activation energies than linear ones of similar weight-average molecular weights, *M_w_* [57]. A value for *E_a_* of 50–70 kJ/mol has been reported for linear PET, whereas a drastic increase to a fivefold higher activation energy, Ea, is reported for long-chain branched PET [56]. In general, it is well-established that LCB polymers have higher values for *E_a_* compared to linear polymers [61,62,63]. The calculated flow activation energy of the investigated samples, Ea, showed a similar trend to that of zero shear viscosity, *η*_0_. The activation energy of virgin PET was 80 KJ/mol, r-PET *E_a_* was 100 KJ/mol, and REX-r-PET Ea was 350 KJ/mol. The increase of up to four times higher Ea is probably due to the presence of LCB. 

Furthermore, the thermorheological behavior provides us with additional insights into the molecular structures of these samples. Figure 9d shows the van Gurp Palmen plot, which is the phase shift, *δ*, as a function of the complex modulus, G*, for virgin PET, r-PET, and REX-r-PET. A thermorheologically simple behavior was observed for virgin PET and r-PET as G*-dependent phase shift values superimposed at different temperatures, meaning that all the relaxation times have the same temperature dependence [64], while REX-r-PET exhibits a systematic split between the curves with the temperature, which identifies the thermorheological complex response. 

The thermorheological complexity of REX-r-PET could also be due to the presence of long-chain branches. A branched structure is assumed to be related with a more pronounced flattening and will eventually lead to an extra bump in the delta versus G* plot, in case the LCB character dominates the behavior [65]. The present result for REX-r-PET does not allow a distinction between different structures, but the minimum observed clearly indicated a second dominating relaxation process that could be attributed to the presence of long-chain branching. In terms of the concentration of LCB, it is reported that LCB-PE metallocene with a sparsely branched structure showed high thermorheological complexity, while LDPE with hyperbranched structures did not [66]. The findings are quite similar to those found for grafted comb and grafted bottlebrush-like LCB-PS [67], where the absence of thermorheological complexity in the PS bottlebrush (number of branches greater than 60) is consistent with the results of LDPE, both having branches statistically distributed along the backbone and, therefore, a similar density of branching points. Considering the high level of thermorheological complexity of REX-r-PET, one could expect that hyperbranched structures are not present. 

#### 3.8.2. The Analysis of the Elongational Rheological Behavior 

**Figure 10:**
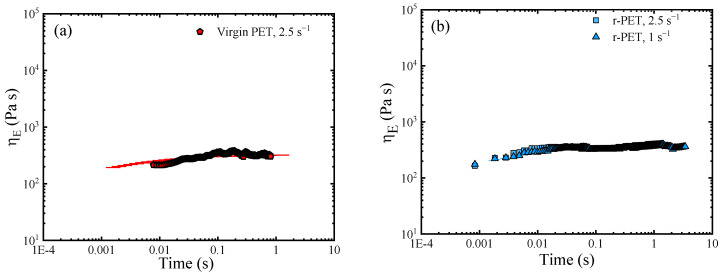

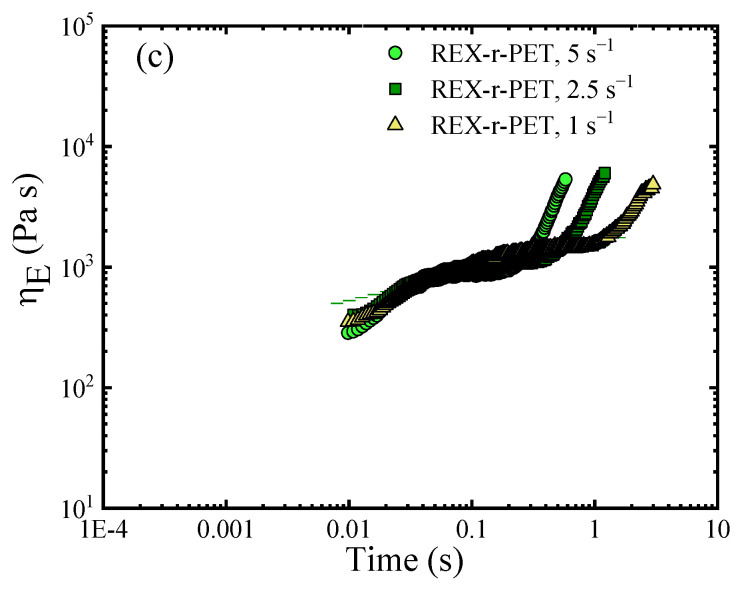
Elongational viscosities for the three PET samples: (**a**) virgin PET, (**b**) r-PET, and (**c**) REX-r-PET.

Elongation rheology tests were also performed to explore the viscoelastic properties of PET. The extensional viscosity curves of virgin and recycled PETs at different elongation rates are represented in Figure 10. For virgin PET (Figure 10a) and r-PET (Figure 10b), the tensile tests were very difficult to perform, because the samples tended to drop during measurements due to their low viscosity. The results confirmed the Newtonian behavior of both samples, as the curves fit the predicted Trouton relationship of three times the complex viscosity data.

The REX-r-PET elongational behavior reflected the molecular structure modification as linear behavior was no longer observed, and strain hardening appeared in the range of the imposed extension rates. This behavior is well-known in typical long-chain branched-dominated rheology, including the response of polymers with architectures ranging from star- and H-shaped polymers to comb and pom-pom structures [68,69,70,71,72,73,74,75,76,77] and has also been reported for LCB Poly (ethylene terephthalate) subjected to reactive treatment with the combination of pyromellitic dianhydride and triglycidyl isocyanurate.

#### 3.8.3. The study of Large Deformation Oscillatory Shear Measurements (LAOS)

Oscillatory shear tests can be divided into two regimes. One regime is a linear viscoelastic response (SAOS) that was addressed in Section 3.8.1, and the second regime is the nonlinear material response (large amplitude oscillatory shear, LAOS)) that will be discussed here. From an experimental point of view, the objective of these nonlinear oscillatory experiments is to investigate the evolution of the nonlinear response with increasing deformation and to quantify the nonlinear material functions. Furthermore, a great effort has been made in the last decades to establish sound relationships between these nonlinearities and the molecular structures of polymers. For that purpose, several quantitative methods have been described for analyzing nonsinusoidal waveforms of shear stresses. Fundamentally, LAOS analytical methods are based upon the principle of Fourier Transform Rheology (FTR). Under shear strain $\gamma \left(t\right)=\gamma_{0}\;\sin\;\left(\omega t\right)$ and a strain rate $\dot{\gamma }\left(t\right)=\gamma_{0}\;\omega \;\cos\;\left(\omega t\right)$, shear stress can be expressed as a Fourier series of elastic and viscous stress.

(9) $$\sigma \left(t;\omega ,\gamma_{0}\right)=\gamma_{0}\sum_{n\;odd}G_{n}^{\prime }\left(\omega ,\gamma_{0}\right)\sin n\omega t+G_{n}^{\prime \prime }\left(\omega ,\gamma_{0}\right)\cos n\omega t$$

where $G_{n}^{\prime }$ and $G_{n}^{\prime \prime }$ are *n*th-order harmonic coefficients. The linear response reduces to the first–order harmonics (*n* = 1), and higher–order harmonic coefficients or phase differences accounts for the nonlinearities, where the relative harmonic intensity ratios $I_{n/1}\equiv \frac{I\left(n\omega \right)}{I\left(\omega \right)}\;\mathrm{or}\;\mathrm{phase}\;\mathrm{angles}\;{\emptyset^{\prime }}_{n}\equiv \emptyset_{n}-n\emptyset_{1}$ are widely used as indicators of nonlinearity. 

Additionally, for every harmonic, an intrinsic nonlinear parameter, Q n(γ0,ω) can be defined in the limit of small-strain amplitudes Q n0(ω). The parameter, which is only frequency dependent, can be defined for every harmonic through the following equation Equation (10):

(10) Q n(γ0, ω)=In/1γ0n−1 with Q n0(ω)=limγ0→0Q n(ω)

Q n0(ω) gives information about the inherent nonlinear material properties of a sample as the trivial scaling $I_{n/1}$α $\gamma_{0}^{n-1}$ is eliminated. 

The intrinsic nonlinearity parameter 3Q, or simply Q, that is derived from the third harmonic, written as in Equation (5), has been reported to be useful in evaluating structural features such as the topology of polymer melts [78,79,80], the droplet size distribution of emulsions [81], and recently, the morphology of polymer blends [82,83].

(11) $$Q\equiv I_{3/1}/\gamma_{0}^{2}\;\mathrm{with}\;\underset{\gamma \to 0}{\lim}Q\equiv Q_{0}$$

To interpret the higher harmonics of a FT rheological series [84], orthogonal stress decomposition is used to separate the nonlinear stress into elastic and viscous contributions based on the symmetry of stress with respect to γ(t) and $\dot{\gamma }$(t). Ewoldt et al. [85] extended this method with the Chebysev polynomials of the first type (*T_n_*), expressing elastic and viscous stresses as:

(12) $$\sigma^{\prime }\left(\gamma /\gamma_{0}\right)=\gamma_{0}\sum_{n,odd}^{\;}e_{n}\left(\omega ,\gamma_{0}\right)T_{n\;}(\gamma /\gamma_{0})$$

(13) $$\sigma \prime \prime \left(\dot{\gamma }/\dot{\gamma_{0}}\right)=\dot{\gamma_{0}}\sum_{n,odd}^{\;}\nu_{n}\left(\omega ,\gamma_{0}\right)T_{n\;}(\dot{\gamma }/\dot{\gamma_{0}})$$

The first-order Chebyshev coefficients (e1 and v1) defined the viscoelastic properties in the linear region (i.e., *e*_1_ *= G′* and *v*_1_ *= G″/ω*). Any deviation from linearity, i.e., the *n* = 3 harmonic, is interpreted depending on the signs of e_3_ and v_3_. A positive third-order contribution results in higher elastic (or viscous) stress at the maximum strain (or strain rate) than is represented by the first-order contribution alone. Thus, depending on the sign of the third-order coefficients, the following physical interpretation can be suggested (see Ewoldt et al. [85] for further details):

(14) $$e_{3}=-G_{3}^{\prime }\{\begin{array}{c} >\;0\;\text{strain-stiffening}\\ =0\;\mathrm{linear}\;\mathrm{elastic}\;\\ <\;0\;\text{strain-softening} \end{array}v_{3}=-\frac{G_{3}^{\prime \prime }}{\omega }\{\begin{array}{c} >\;0\;\text{shear-thickening}\\ =0\;\mathrm{linear}\;\mathrm{viscous}\;\\ <\;0\;\text{shear-thinning} \end{array}$$

**Figure 11:**
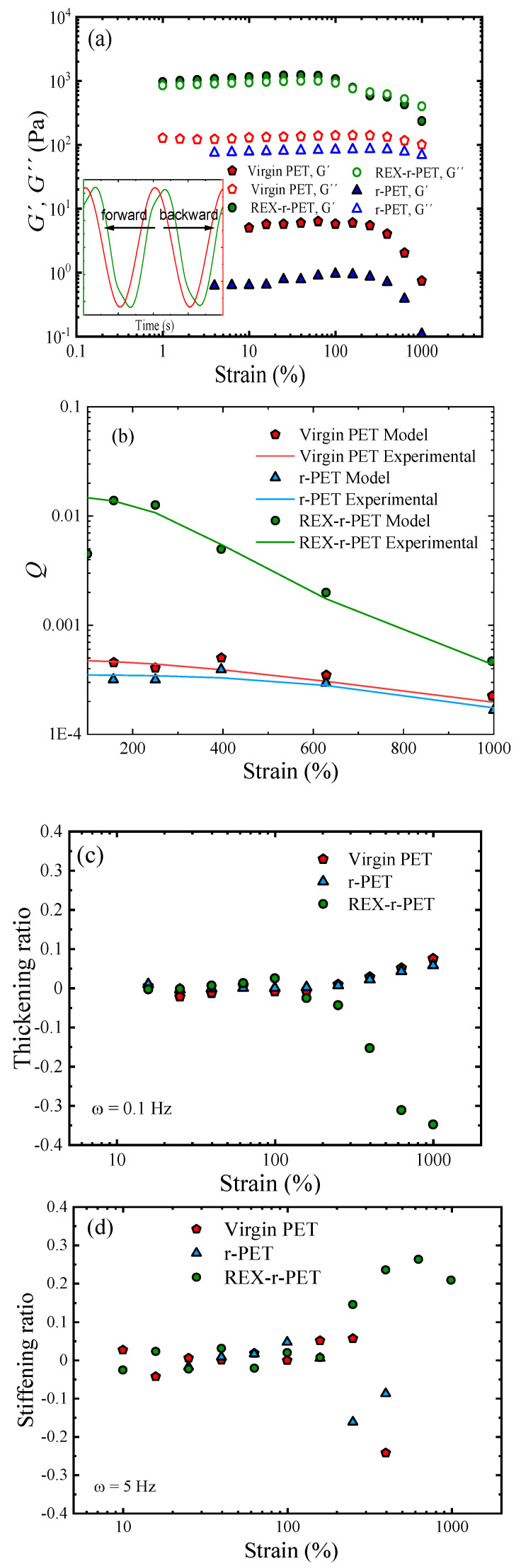
Results for the LAOS nonlinear oscillatory test. (**a**) Evolution of the storage modulus, *G*1′ and *G*1″, as a function of the strain obtained at a constant frequency of 0.1 Hz. We focus on the stress curve, which corresponds to 200% of the applied strain for virgin PET and REX-r-PET. (**b**) The intrinsic nonlinearity *Q*_0_ of three investigated samples obtained in the MAOS regime. (**c**) Evolution of the shear-thickening ratio with strain at 0.1 Hz. (**d**) Evolution of the shear-stiffening ratio with strain at 5 Hz.

Nonlinear responses obtained at a constant frequency of 0.1 Hz and T = 260 °C of virgin PET and REX-r-PET are shown in Figure 11a. At a small *γ*_0_, in the linear region, *G*′ and *G*′′ remain constant values, but, as the applied amplitude of strain is increased from small to large, a transition between the linear and nonlinear regime is observed so that in the nonlinear regime, both virgin PET and REX-r-PET, display moduli that decrease with the increasing strain (Figure 11a). It is interesting to note that the stress patterns of the molten virgin PET and r-PET differ from that of the REX-r-PET (see insert in Figure 11a, which corresponds to the stress signals at 200% of the strain; r-PET data are not included for clarity). 

As observed (in the Figure 11a inset), the unmodified virgin PET displays a weak distortion, whereas REX-r-PET displays a “backward-tilted” shape stress. The distorted directions are considered to be related to specific polymer structures. Previous studies revealed that “forward-tilted stress” tends to appear in the case of polymer melts and solutions with a linear chain structure [86,87], whereas “backward-tilted stress” was reported for suspensions and polymer melts with branched chains. This behavior is generally attributed to the effect of branched structures during the flow alignment of polymer chains occurring at the larger strains. Branching is considered an obstacle and leads to an extra resistance to the flow. As a result, a stress shoulder appears at higher times—that is, the stress tilts backwards, delayed with respect to the symmetry axis. Interestingly enough, the distorted directions were reported to be related to the relative magnitudes of *e*_3_/*e*_1_ and *v*_3_/*v*_1_ in the nonlinear responses in the case of filled and vulcanized polyisoprene, respectively [88]. Therefore, as a first approach, nonlinearity is very sensitive to the different structures of these materials. To further distinguish the differences in the topological structure of the PET samples, the analysis of the nonlinear region is divided in terms of the MAOS (medium amplitudes oscillatory shear) and LAOS (large amplitudes oscillatory shear) regimes. 

Under MAOS, using the FT rheology method, the third-harmonic intensity normalized by the first-harmonic intensity (*I*_3/1_) can be used as a representative nonlinear parameter, helpful to detect the boundary of linear-to-nonlinear transition. In this regime (50–300%), the third-harmonic intensity is the only higher harmonic contribution, and the parameter *I*_3/1_ scaled quadratically with the strain amplitude as expected [89]. According to the experimental and theoretical findings, Hyun and Wilhelm [90] suggested that the intrinsic nonlinearity *Q*_0_ in the MAOS regime can be applied to detect different polymer architectures, e.g., linear, 3-arm star, comb with many branches, and long-chain branching architectures. Figure 11b clearly differentiates the evolution of the *Q* parameter with the strain amplitude for the three samples: virgin PET, r-PET, and REX-r-PET. *Q* has a constant value (*Q*_0_) at relatively small strain amplitudes, while it becomes a function of the strain at larger strain amplitudes. At the investigated frequency of 0.1 Hz, the *Q*_0_ value for REX-r-PET is much higher than the values of virgin PET and r-PET, for which *Q*_0_ is very similar. Ahirwal et al. [91] obtained results for the branched PP and branched PE comparable to those obtained for the PET samples here. They found that the *Q*_0_ parameter increased monotonically as a function of the long-chain branched PP weight fraction in the PP blends.

The structural differences of PET samples can be more evidently characterized under LAOS (in our case, from 300 to 900%). By decomposing the nonlinear stress waveforms based on symmetry arguments and using a Chebyshev polynomial analysis, the contribution of higher-order harmonics can be useful to gain advanced understanding in terms of the elastic and viscous nonlinearities described, respectively, by the intracycle strain stiffening (or softening) and intracycle strain rate thickening (or thinning) indices. The analysis could find application in the evaluation of molecular architecture and branching characteristics of polymer melts. 

Figure 11c,d shows the comparisons among intracycle nonlinear coefficients of the investigated samples. The viscous nonlinear thickening ratio (Figure 11c), ν_3_, and the elastic nonlinear stiffening ratio (Figure 11d), *ε*_3_, defined by Equation (11), are plotted as a function of the applied strain. On the one hand, viscous nonlinearity between the samples was found to differ especially at the lower frequencies. 

Figure 11c shows the strain dependence of the thickening ratio obtained at 0.1 Hz for the three samples. The virgin PET and r-PET samples were characterized by quasilinear behavior, whereas REX-r-PET was characterized by strong strain rate thinning (ν_3_ < 0). On the other hand, elastic nonlinearity was found to be more sensitive to high frequencies, because REX-r-PET showed the strain thickening (*e*_3_ > 0) increasing with the frequency (frequency effect not shown to avoid data overlapping). 

Figure 11d shows the different stiffening ratios obtained at 5 Hz for the three samples. As a general trend, viscous and elastic nonlinear behavior were analogous to the response previously described in shear and elongational rheological tests. Under shear, REX-r-PET showed pseudoplastic behavior in contrast to the quasi-Newtonian response obtained for virgin PET and r-PET. Additionally, under melt elongational experiments, REX-r-PET showed typical strain hardening, as the sample hardens when the strain increases at a constant strain rate, a behavior not present in virgin PET and r-PET. Similarly, during the LAOS test, the stiffening behavior of PET REX can be understood when considering the ability of the branched structure to stretch during the oscillatory flow and re-stretch in the reverse direction.

### 3.9. Mechanical Properties 

One of these disadvantages of the mechanical recycling of PET is that chain scission decreases the molecular weight and intrinsic viscosity, as observed in the previous section. These results could affect the mechanical properties. 

**Figure 12:**
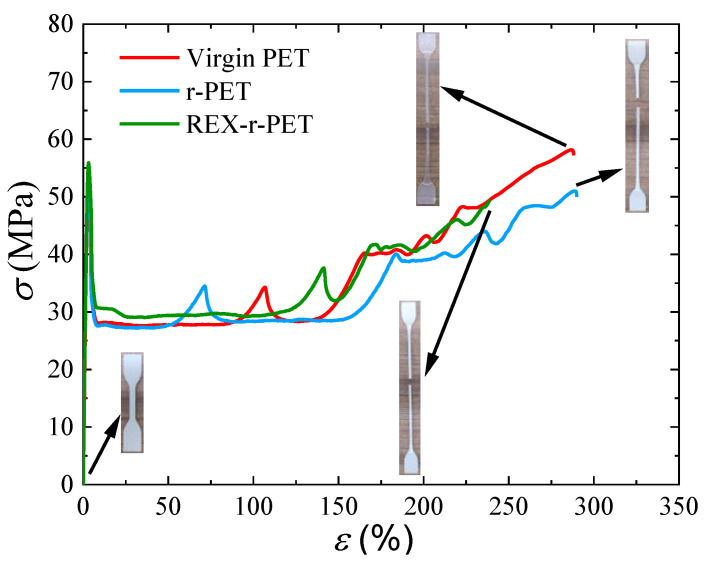
Stress–strain curves for virgin PET, r-PET, and REX-r-PET.

**Figure 13:**
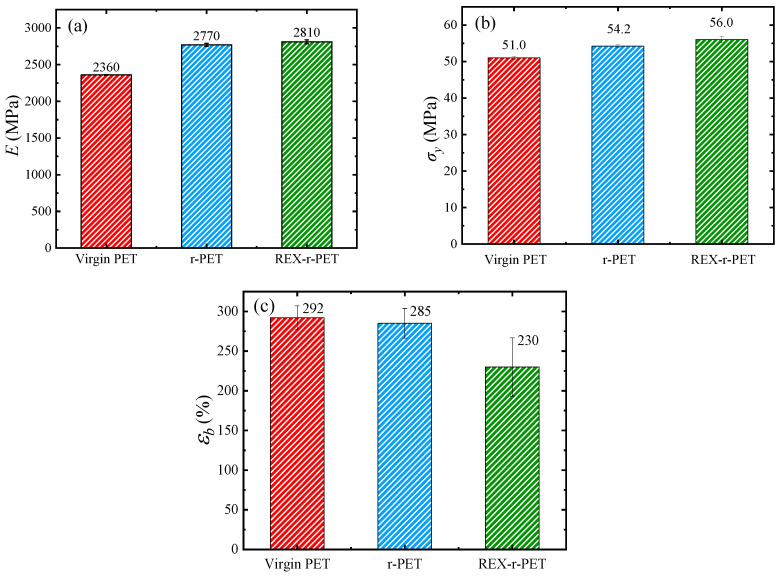
(**a**) Young’s modulus, (**b**) yield stress, and (**c**) strain-at-break values of virgin PET, r-PET, and REX-r-PET.

Figure 12 shows typical stress–strain curves of virgin PET, r-PET, and REX-r-PET. The Young’s modulus, yield strength, and strain-at-break values obtained from these curves are shown in Figure 13. As can be seen in Figure 12, recycling does not significantly affect the overall tensile behavior of the material, as both r-PET and REX-r-PET showed very ductile behaviors, similar to that of virgin PET, as well as similar cold-drawing and strain-hardening behaviors.

With respect to the Young’s modulus, as can be observed in Figure 13a, recycled materials showed higher values than virgin PET. Among the usual factors affecting the Young’s modulus of polymeric materials, three of them must be considered in this case: (1) changes in the molecular weight of the polymeric matrix, (2) changes in the crystallinity as a result of the different molecular weights or the presence of inorganic fillers, and (3) the stiffening effect of the TiO_2_ particles in both recycled materials.

**Figure 14:**
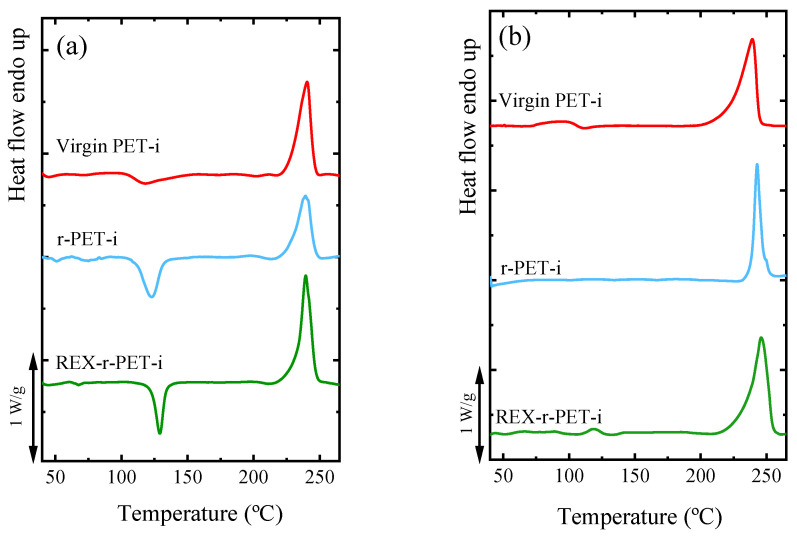
DSC scans of PET-injected samples: first heating runs from (**a**) the surface and from (**b**) the core of the injected specimens. Heating rates were 20 °C/min in both cases.

Figure 14 and Table 5 show the DSC results of as-molded virgin PET, r-PET, and REX-r-PET both at the surface and the core of the injection-molded specimens. As usual, in these kinds of semicrystalline materials with slow crystallization rates, an increasing gradient of crystallinity from the surface to the core was observed. However, when the three materials were compared, the differences in *X_c_* were not far from the experimental error of the measurements, which, in these cases, was approximately 10–20%. Thus, crystallinity must be ruled out as the reason for the changes observed in the Young’s modulus. With respect to the other two effects, on the one hand, it is well-known that molecular weight decreases may lead or not to lower Young’s modulus values, depending on the range of the decrease [20,92]. On the other hand, it is also well-known that nanofillers improve the low-strain mechanical properties of most polymeric matrices [93] and, specifically, of PET [50,94]. Thus, the significantly higher Young’s modulus value of the two recycled materials, given their lower molecular weight with respect to the virgin PET, must be related to the presence of the rigid TiO_2_ particles, this effect prevailing over that, if any, of the reduction in the molecular weight. The small differences observed between the two recycled materials agree with the changes in the molecular weight, but the values are within the experimental error of the measurement, and thus, the differences are hardly significant. The higher molecular weight of REX-r-PET with respect to r-PET is the result of chain extension reactions caused by reactive extrusion carried out in the presence of a chain extender [19,20,34]. REX-r-PET is characterized not only by a higher molecular weight than r-PET but, also, by the presence of long-chain branches, as demonstrated above by both the SAOS and LAOS measurements.

As shown in Figure 13b, the behavior of the yield stress was similar to that of Young’s modulus: the recycled materials showed higher yield stress values compared to the virgin PET, and the highest value corresponded to REX-r-PET. In previous works, it has been observed that the yield stress usually follows the same trend as the Young’s modulus [95,96,97,98,99,100] as it does in the present work.

Figure 13c shows the strain at break values of virgin PET, r-PET, and REX-r-PET. As mentioned above, it is remarkable that both recycled materials show very high elongation at break values, with the strain at break values over 200%. Still, virgin PET is the one that shows the highest elongation at break values. The above-mentioned gradient of crystallinity degree from the surface to the core in the three materials can help to explain this high deformation ability. When the three materials are compared, again, the two main factors that are expected to have a negative effect on ductility are, on the one hand, the presence of nanoparticles in the matrix and, on the other hand, the decrease in molecular weight [101,102,103]. A lower ductility of the recycled materials is, indeed, to be expected as a consequence of the presence of nanoparticles, which is attributed to restrictions in the mobility of the matrix chains caused by the nanoparticles that promote a fracture. Furthermore, lower molecular weights lead to a poorer strain at the break values. When the two recycled materials are compared, it is observed that the ductility of REX-r-PET is significantly lower than that of r-PET. In this case, given that the TiO_2_ nanoparticle concentration is the same in both recycled materials and the molecular weight is lower in r-PET, the branched chain structure of REX-r-PET could be the reason for the observed ductility decrease in REX-r-PET in comparison with the other samples, according to the previously shown rheological results.

With respect to the toughness, Table 6 shows the impact strength values of virgin PET, r-PET, and REX-r-PET. It is observed that the impact strength is lower in the recycled materials when compared to the virgin material. As in the case of ductility, the presence of nanoparticles [104,105,106,107] and the lowered molecular weight [108] of both r-PET and REX-r-PET with respect to virgin PET have a negative effect on its impact properties. The difference observed between r-PET and REX-r-PET is not significant, as it is within the experimental error of the measurements. In any case, the impact resistance values are, in general, low, as usually happens with notch-sensitive materials [109,110,111].

## 4. Conclusions

In this work, post-consumer recycled opaque PET was homogenized by extrusion (r-PET) and, also, modified by reactively extruding the material with Joncryl (REX-r-PET). The reactive extrusion changed the molecular structure of the originally linear r-PET by introducing long-chain branches in the material and increasing the average molecular weight of the material. 

Isothermal crystallization studies demonstrated that the introduction of long-chain branches decreased both the nucleation and growth rates of REX-r-PET in comparison with r-PET.

According to the rheological characterization, the linear and nonlinear viscoelasticity, as well as the elongational behavior, were profoundly affected by the reactive extrusion process. Virgin PET and r-PET showed a rapid relaxation (relaxation time less than one second) related to its linear structure, and a Newtonian response was found under shear and elongational deformation. REX-r-PET was characterized by a slower relaxation process with enhanced pseudoplasticity, thermorheology, and elongational strain-hardening behavior, which indicated the formation of longer molecules and, most probably, long-chain branches during reactive extrusion. Correspondingly, the nonlinear viscoelastic response was also clearly enlarged. It is shown that both the increase in the intrinsic nonlinearity *Q*_0_ value determined in the MAOS regime, as well as the viscous and elastic nonlinearity in terms of strain-thinning and strain-stiffening behaviours analyzed in the LAOS regime, could serve as sensitive indicators of the structural changes induced by the addition of an extender/branching Joncryl additive.

The main factors affecting the mechanical properties of the recycled materials with respect to virgin PET are the decrease in molecular weight, the presence of TiO_2_ nanoparticles, and in the case of REX-r-PET, the presence of long-chain branching. As a consequence of the presence of TiO_2_ nanoparticles, low-strain mechanical properties (i.e., Young’s modulus and yield stress) increase with respect to the virgin PET both in r-PET and REX-r-PET, which are even higher in the latter, likely due to the long-chain branched matrix. On the contrary, high-strain mechanical properties (i.e., ductility and impact strength) decrease in the recycled materials with respect to the virgin PET for the same reasons (i.e., lower molecular weights, presence of nanoparticles, and long-chain branching in REX-r-PET). However, it is remarkable that, even after undergoing a recycling process (either reactive of nonreactive), the materials remain very ductile. 

The reactive extrusion process is a suitable way to recycle opaque PET into a material with enhanced rheological properties (thanks to the production of chain extension and long-chain branches) with mechanical properties that are comparable to those of a typical commercially available PET sample employed for bottle manufacturing.

## Figures and Tables

**Table 1:** Intrinsic viscosity values of the PET samples.

Material	[*η*] * (dL/g)	*M_w_* (kDa)	*M_n_* (kDa)
Virgin PET	0.702	45.0	22.5
r-PET	0.526	27.1	13.6
REX-r-PET	0.626	37.3	18.7

* Where to = 42.3 s, *K*(*M_n_*) = 1.166 × 10^−3^, *K(M_w_*) = 7.44 × 10^−4^, and α = 0.648.

**Table 2:** Thermal parameters obtained by DTGA.

Sample	*T*_*d*,2%_	*T_d_*_1_ (°C)	*T_d_*_2_ (°C)	Residue at 800 °C (%)
Virgin PET	326	432	560.9	0
r-PET	311	430	561.0	2.40 ± 0.08
REX-r-PET	324	432	561.0	2.62 ± 0.21

**Table 3:** Thermal properties of the PET samples. All values were obtained from the DSC scans shown in Figure 3.

	First Heating		Cooling			Second Heating		
	*T_m_* (°C)	Δ*H_m_* (J/g)	*X_c_* (%)	*T_c_* (°C)	Δ*H_c_* (J/g)	*T_m_* (°C)	Δ*H_m_* (J/g)	*X_c_* (%)
Virgin PET	241.0	34	24	-	-	243.5 179.8 (T_cc_)	31 14 (ΔH_cc_)	11
r-PET	242.9	41	30	183.6	35	239.9	36	27
REX-r-PET	241.5	40	29	178.3	30	237.9	32	23

**Table 4:** Values obtained by fitting the L–H theory to the experimental DSC overall crystallization data. Parameter proportional to the energy barrier for the secondary nucleation ($K_{g}^{\tau }$ ), fold surface energy (*σ_e_*), and work done by the chain to perform a fold (*q*). *R^2^* is the correlation coefficient for the fitting of the L–H model Equation (4).

Sample Name	$K_{g}^{\tau }$ (K^2^)	*σ* (erg/cm^2^)	*σ_e_* (erg/cm^2^)	*q* (erg)	*R* ^2^
Virgin PET	3.81E^+05^	9.03	275	1.01E^−12^	0.992
r-PET	3.84E^+05^	9.03	276	1.02E^−12^	0.978
REX-r-PET	5.91E^+05^	9.03	426	1.57E^−12^	0.991

**Table 5:** Thermal properties of the PET-injected samples. All values were obtained from the DSC first heating scans shown in Figure 14.

	Surface					Core		
Materials	*T_m_* (°C)	Δ*H_m_* (J/g)	*T_cc_* (°C)	Δ*H_cc_* (J/g)	*X_c_* (%)	*T_m_* (°C)	Δ*H_m_* (J/g)	*X_c_* (%)
Virgin PET-i	240.9	29	117.1	3.5	18	240.2	39	29
r-PET-i	239.4	24	123.0	9.4	11	244.2	32	23
REX-r-PET-i	239.2	27	129.1	10.3	12	245.9	41	30

**Table 6:** The impact strength values of virgin PET, r-PET, and REX-r-PET.

Sample	Impact Strength (J/m)
Virgin PET	27.6 ± 0.7
r-PET	21.5 ± 3.1
REX-r-PET	24.3 ± 0.4

## Data Availability

The data presented in this study are available on request from the corresponding author.

## Supplementary Materials

The following are available online at https://www.mdpi.com/article/10.3390/polym13203531/s1: Figure S1: DTGA. Figure S2. An example of Avrami plots of the data obtained during the crystallization of r-PET-O at 200 °C. (**a**) Avrami plot of the experimental data obtained during crystallization. (**b**) Normalized heat flow experimental data during crystallization compared to the data predicted data by the Avrami model. Table S1: Parameters obtained from fitting the DSC data presented in Figure S1 to the Avrami model.
