# Supplementary material for: Structure and Properties of Reactively Extruded Opaque Post-Consumer Recycled PET
Source: Polymers (Basel). 2021 Oct 14;13(20):3531. doi: 10.3390/polym13203531 (PMC8540998; doi:10.3390/polym13203531)
Supplement: Supplementary file 1 [file polymers-13-03531-s001.zip › polymers-1399153-supplementary.pdf]

**Supplementary material**

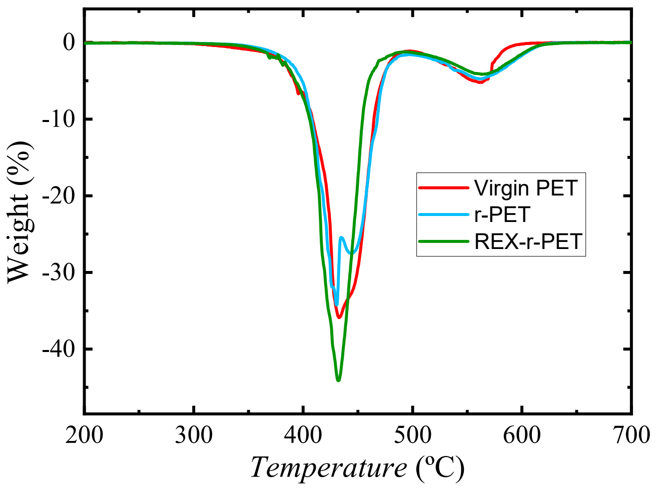


**Figure S1**. DTGA.

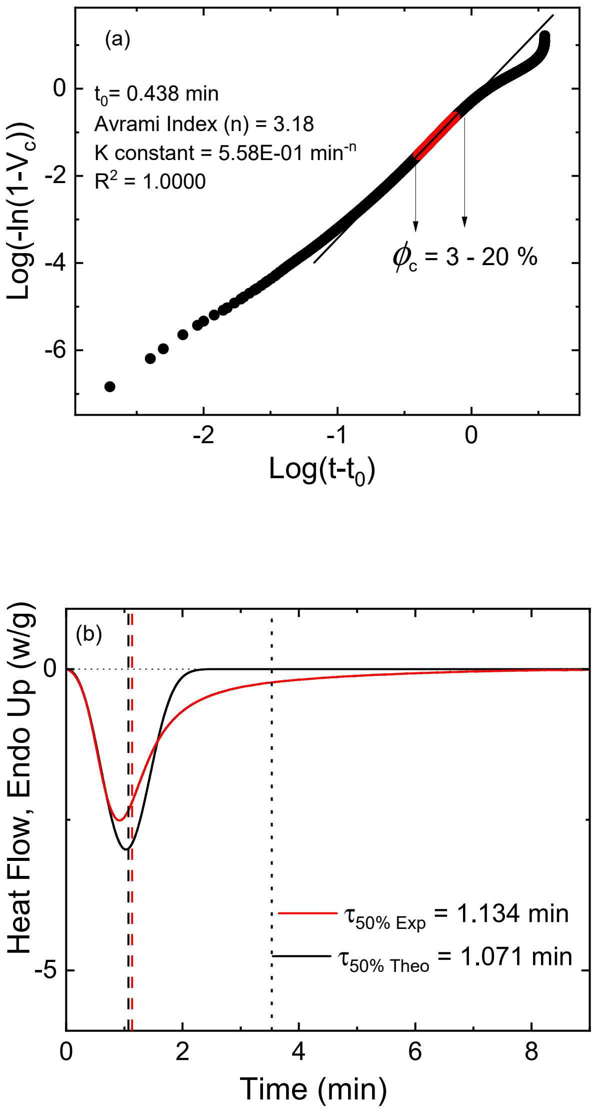


**Figure S2**. An example of Avrami plots of the data obtained during crystallization of r-PET-O at 200 ºC. (a) Avrami plot of the experimental data obtained during crystallization. (b) Normalized heat flow experimental data during crystallization compared to the data predicted data by the Avrami model.

**Table S1**. Parameters obtained from fitting the DSC data presented in Figure SI-1 to the Avrami model.

| $T_c$ (ºC) | $t_0$ (min) | $n$ | $K$ (min-$^{n}$) | $K_{1/n}$ | $R$ | $t_{50\%\ exp}$ (min) | $t_{0\%\ Theo}$ (min) | $1/\ t_{50\%\ exp}$ (1/min) |
|---|---|---|---|---|---|---|---|---|
| 200 | 0.345 | 2.89 | 0.853 | 0.6316 | 1 | 0.931 | 0.957 | 1.07411 |
